# Retention Load Values of Telescopic Crowns Made of Y-TZP and CoCr with Y-TZP Secondary Crowns: Impact of Different Taper Angles

**DOI:** 10.3390/ma9050354

**Published:** 2016-05-11

**Authors:** Susanne Merk, Christina Wagner, Veronika Stock, Patrick R. Schmidlin, Malgorzata Roos, Marlis Eichberger, Bogna Stawarczyk

**Affiliations:** 1 Department of Prosthodontics, Dental School, Ludwig-Maximilians-University Munich, Goethestrasse 70, Munich 80336, Germany; merk.susanne@googlemail.com (S.M.); chrissy.wagner@gmx.net (C.W.); stock.veronika@web.de (V.S.); marlis.eichberger@med.uni-muenchen.de (M.E.); 2 Clinic of Preventive Dentistry, Periodontology and Cariology, Center of Dental Medicine, University of Zurich, Plattenstrasse 11, Zurich 8032, Switzerland; patrick.schmidlin@zzm.uzh.ch; 3 Division of Biostatistics, Epidemiology Biostatistics and Prevention Institute, University of Zurich, Hirschengraben 84, Zurich 8001, Switzerland; mroos@ifspm.uzh.ch

**Keywords:** Y-TZP, telescopic crowns, CAD/CAM, retention load, electroforming

## Abstract

This study aimed to examine and compare the retention load values (RL) of different telescopic crown assemblies (Y-TZP and CoCr primary crowns with electroformed and Y-TZP secondary crowns each) with three different taper angles (0°, 1° and 2°). Thirty Y-TZP primary crowns with electroformed gold copings (Z/G group) and Y-TZP secondary crowns (Z/Z group) and 30 CoCr primary crowns with electroformed gold copings (C/G group) and Y-TZP secondary crowns (C/Z group), each with taper angles of 0°, 1° and 2°, were fabricated, respectively. With the exception of the electroformed gold copings, all specimens were Computer-Aided-Design/Computer-Aided-Manufacturing (CAD/CAM)-milled, then sintered and afterwards manually adapted. In order to stabilize the gold copings, they were fixed in a tertiary structure. The secondary crowns were constructed with a hook, which ensured self-alignment with an upper chain. Afterwards, 20 pull-off test cycles were performed in a universal testing machine under artificial saliva and after weighing the secondary crowns with a 5 kg object for 20 s. Data were analyzed by one-way and two-way Analysis of Variance (ANOVA). C/Z with 1° showed higher (*p* = 0.009) RL than 0° and 2° tapers. C/G at 1° also showed higher (*p* = 0.001) RL than at tapers of 0° and 2°. Z/G and C/G at 0° showed lower RL than Z/Z and C/Z (*p* < 0.001). Primary crowns had no impact on the 0° group. Z/G showed lower RL as compared to C/Z within the 1° group (*p* = 0.007) and Z/Z in the 2° group (*p* = 0.006). The primary crown material had no influence on RL. Electroformed copings showed lower RL. Further investigations for 1° as well as for the long-term performance after thermomechanical aging are necessary.

## 1. Introduction

Zirconia (ZrO_2_), a ceramic material with great potential, has been used in different medical applications for quite some time [1]. Especially in orthopedic surgery, ceramic materials have proven themselves for a long time [1,2]; the reason they are being used is based on their excellent mechanical properties: temperature stability, strength and resistance to acids and alkalis [3]. In addition, dental medicine has recognized a wide range of other applications of the material in the field: It is used for crowns [4], fixed dental prostheses (FDPs) [5], implant abutments [6], removable partial dentures (RDPs) [7], *etc*. Consequently, ZrO_2_ has become a widely used material in dentistry [4,6,8,9,10,11] because of its excellent properties such as biocompatibility [1,2], mechanical strength, aesthetic appearance and chemical resistance [9,10,11], and the material has also proven outstanding biocompatibility in clinical studies [1,2]. Furthermore, it can be polished or ceramically veneered [10,11], which is also very important in the field of esthetic dentistry. The physical properties of the ZrO_2_ material can be improved by stabilization with the metallic oxide yttrium (Y_2_O_3_) [1]. The resulting yttrium-stabilized tetragonal zirconium oxide (Y-TZP) shows even better mechanical properties than other zirconia oxides [2], which is a result of the crystalline modification from a tetragonal (T) to monoclinic (M) arrangement [2]. This T-M transition occurs after a cracking which creates energy to seal the crack by expansion [2].

Regardless of the stabilizing procedure, Y-TZP proved itself in long-term studies. Two recent clinical studies on single crowns yielded good success rates [4,8]. Three-unit FDPs also presented a survival rate similar to conventional FDPs [5] after 10 years. In a seven-year study, Kolgeci and co-workers observed that Y-TZP-based prostheses are clinically successful on dental implants as well [12]. This was corroborated by another 10-year clinical study [13].

Y-TZP was also used and studied as a material for telescopic crown systems [7,9]. Most of these studies assessed assemblies with Y-TZP primary crowns and secondary crowns of a different material, especially gold alloy. In this context, the combination of a ZrO_2_ primary crown with a galvanic-formed gold coping showed more a predictable and less excursive retention load than conventionally cast telescopic crowns [9,14]. However, primary and secondary crown assemblies totally made of Y-TZP have not been thoroughly investigated so far. To the authors’ best knowledge there is only one investigation of such homogenous Y-TZP joints [7].

The electroforming process can achieve a very precise fit for the primary crown onto the secondary crown [15], which is a result of the manufacturing process. A thin layer of silver conductive lacquer applied on the outer surface of the primary crown and the automatically running electroplating process create the precise secondary coping [15]. The gold coping produced demands no adjustment like conventionally cast ones do [15]. For these telescopic joints, hydrodynamic effects especially and the adhesion of liquids provide the retention load [15,16]. The galvanic copings are made of 99.9% pure gold [16] with an elastic modulus of 78.5 GPa [17]. Another much stiffer metal with an elastic modulus similar to that of Y-TZP can be used for primary crowns, namely cobalt-chromium alloy (CoCr). According to the manufacturer's specifications, the value is 204 GPa for Y-TZP and 200 GPa or greater for CoCr. The latter one, assembled with the electroformed secondary crown, was investigated by Engels and co-workers [14]. This study showed that CoCr had actually higher retention load values as compared to gold or ZrO_2_ crowns.

Even if Besimo *et al.* observed no significant influence of the primary crown material [18], there has been a contrary outcome documented. In this investigation the surface roughness of the primary crowns affected the retention load of electroformed assemblies [19]. In recent studies it was assumed that different hardness levels [9] and the surface treatments such as polishing [16] may have an impact on the retention load values.

The aim of the present study was to determine the retention load values of differently assembled telescopic crown systems:

1. Y-TZP primary crown with a secondary crown made of Y-TZP (Z/Z) and electroformed copings (Z/G);
2. CoCr primary crown with a secondary crown made of Y-TZP (C/Z) and electroformed copings (C/G).

Each assembly was created with three different taper angles (0°/1°/2°).

The first null hypothesis was that the taper angle will show no influence on the retention loads. The second null hypothesis was that the material of the primary crown has no impact on the retention load.

## 2. Materials and Methods

**Figure 1:**
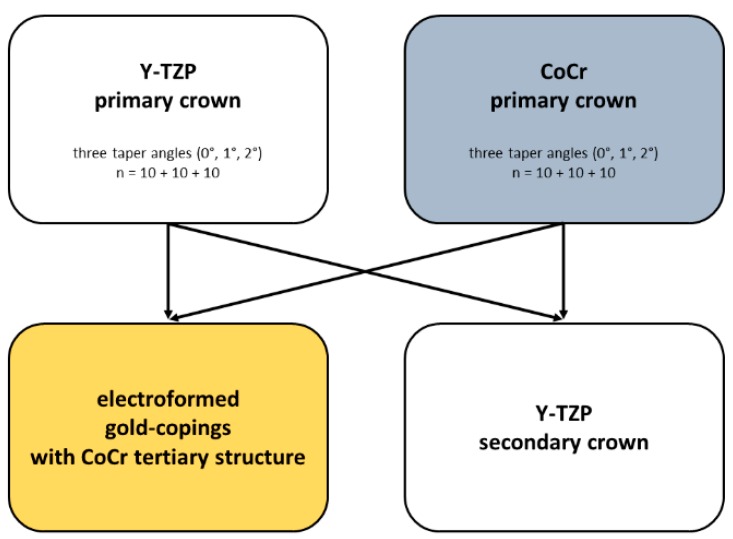
Treatment groups and combination of the telescopic crown assemblies.

This study determined the maximum retention load values of 120 telescopic crowns (Figure 1). The primary crowns were made from:

1. Yttrium-stabilized tetragonal zirconium dioxide polycrystals (Y-TZP) (Ceramill ZI 71; AmannGirrbach AG, Koblach, Austria, LOT: 1303002) or;
2. Cobalt-chromium alloy (CoCr) (Ceramill Sintron 71 16 millimeter; AmannGirrbach AG, LOT: 1303045).

The secondary crowns were made from Y-TZP (CERAPP Zirkon Blank; Ingenieurbüro Sax IBS, Kaisersesch, Germany, LOT: 3YZ-L34-1106313-W-007-18-009) and electroformed gold copings fixed in a CoCr tertiary structure (Ceramill Sintron; AmannGirrbach AG, LOT: 1402005).

### 2.1. Retention Load Measurement

Each combination of primary and secondary crowns was tested in 20 cycles by a pull-off test under the same conditions, *i.e.*, moistening of the primary crown with artificial saliva (Glandosane, No. 9235461109, cell pharm, Bad Vilbel, Germany) and weighing the secondary crown with a weight of 5 kg for 20 s. For measuring the retention load, specimens were placed and fixed in a universal testing device (Zwick 1445, Zwick, Ulm, Germany). For this purpose, the secondary crowns were provided with a retaining jig that was connected to a hook. The latter and its upper chain were part of the pull-off test set-up and ensured self-aligning. The tests were performed with a cross head speed of 50 mm/min.

### 2.2. Fabrication of Primary Crowns

**Figure 2:**
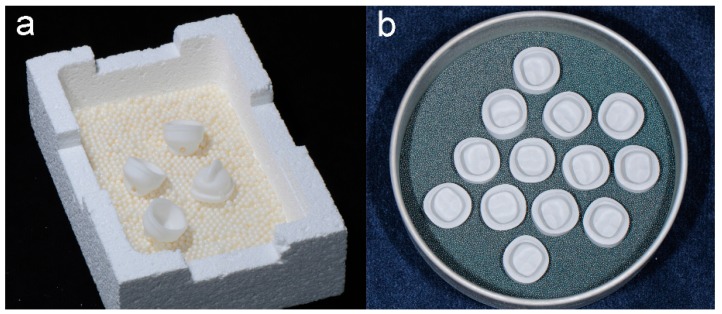
Comparison of the sintering procedures: Sinter support structure with Y-TZP secondary crowns (**a**); and CoCr primary crowns (**b**).

As a basis for the abutments a prepared plastic model tooth was duplicated with a silicone mold (Adisil blau 9:1, Siladent, Goslar, Germany). Sixty wax abutments (Milling- & Universal Wax blue; GEBDI, Engen, Germany) were transferred into a base metal alloy (Remanium GM800+; Dentaurum, Ispringen, Germany, LOT: 936) using the conventional casting method. Afterwards, these metals abutments were scanned (Ceramill map 300, AmannGirrbach AG) and six different constructions of primary crowns were designed: three for zirconia primary crowns and three for cobalt-chromium primary crowns with three tapers each, a 0° with chamfer preparation and a 1° and 2° with tangential ending, respectively (Ceramill mind, AmannGirrbach AG). Each design was CAD/CAM milled 10 times with a milling machine (Ceramill Motion 2 System, AmannGirrbach AG) from chalky Y-TZP (Ceramill ZI 71, AmannGirrbach AG, LOT: 1303002) and cobalt-chromium-molybdenum alloy blanks (Ceramill Sintron 71 16 millimeter; AmannGirrbach AG, LOT: 1303045). In summary, 30 Y-TZP primary crowns and 30 CoCr primary crowns (Figure 2b) were sintered.

#### 2.2.1. Y-TZP Primary Crowns

The sintering process was performed in a sintering furnace according to the manufacturer’s recommendations (Ceramill therm, AmannGirrbach AG). After adhesive placement using a self-adhesive resin cement (RelyX Unicem 2, 3M ESPE, Seefeld, Germany, LOT: 509981), the sintered Y-TZP crowns were mounted in a socket in their insertion direction. Afterwards, the tapers were manually adapted with a water-cooled turbine (W & H Perfecta 900, W & H Dentalwerk Bürmoos GmbH, Bürmoos, Austria) and fixed in a parallelometer (F4 basic, DeguDent, Hanau, Germany). For this purpose, diamond burs (Ceramic Art Set 4371/4369, ZR374M/F, Komet Dental GmbH & Co. KG, Lemgo, Germany) with three corresponding grit sizes (151 µm/107 µm/46 µm) for 0°, 1° and 2° tapers were used as recommended in the literature. For polishing, a three-step silicone polishing system (Ceramic Art Set 4371, Komet Dental GmbH & Co. KG) was applied with round brushes and polishing paste (Komet Dental GmbH & Co. KG, REF: 9638900190; YETI DIA-GLACE; YETI Dentalprodukte GmbH, Engen, Germany, Pat. 3832085.1).

#### 2.2.2. CoCr Primary Crowns

According to the manufacturer’s recommendation, the chalky cobalt-chromium crowns were sintered in a protective atmosphere with argon gas (Ceramill Argotherm, AmannGirrbach AG). After being air-abraided with 110 µm mean alumina particles with 2 bar (basic Quattro IS; Renfert GmbH, Korox 110, Bego GmbH & Co. KG, LOT: 14878430513), the primary crowns were cemented and mounted in a socket similar to the Y-TZP procedure as mentioned above. They were adapted with a hand piece fixed in a parallelometer and cross-cut burs with appropriate tapers (tungsten carbide burs, Komet Dental GmbH & Co. KG, LOT: 042830) and finished with polishing brushes and paste (Komet Dental GmbH & Co. KG, LOT: 226983; Abraso-Starglanz asg, bredent GmbH Co. KG, Senden, Germany, REF: 52000163).

### 2.3. Fabrication of Secondary Crowns

#### 2.3.1. Y-TZP Secondary Crowns

The 60 polished primary crowns (30 × Y-TZP + 30 × CoCr) were scanned (Arti-Spray, white, BK 285, Dr. Jean Bausch GmbH & Co. KG, Cologne, Germany; Ceramill map 300, AmannGirrbach AG) and respective constructions were designed (N = 10 per taper), *i.e.*, 30 constructions on Y-TZP primary crowns and 30 on cobalt-chromium primary crowns, respectively. Afterwards, these 60 Y-TZP secondary crowns were milled from chalky Y-TZP blanks (CERAPP Zirkon Blank; ZENO Tec System, ZENO 4030 M1, Wieland Dental GmbH & Co. KG, Pforzheim, Germany). After the sintering process (Figure 2a), the fitting of the secondary crowns to their primary crowns was adapted with diamond burs (ZR 8850, Komet Dental GmbH & Co. KG) and the polishing process was handled similarly to the Y-TZP primary crowns.

#### 2.3.2. Electroformed Secondary Crowns

The other 60 secondary crowns worked with a galvanic formed inner coping, produced in a galvanic device (Hafner HF 600.3; C. Hafner GmbH & Co. KG, Pforzheim, Germany) in an electroforming gold bath containing electrolyte solution (Helioform H Electrolyte; C. Hafner GmbH & Co. KG, LOT: 00433724) and the related gold solution (Helioform H Concentrate, C. Hafner GmbH & Co. KG, LOT: 0043468). The finished copings were mounted (AGC Cem Automix system, Wieland Dental GmbH & Co. KG, LOT: 697720) into a superstructure to enhance the thin gold copings and to carry out the pull-off tests.

**Figure 3:**
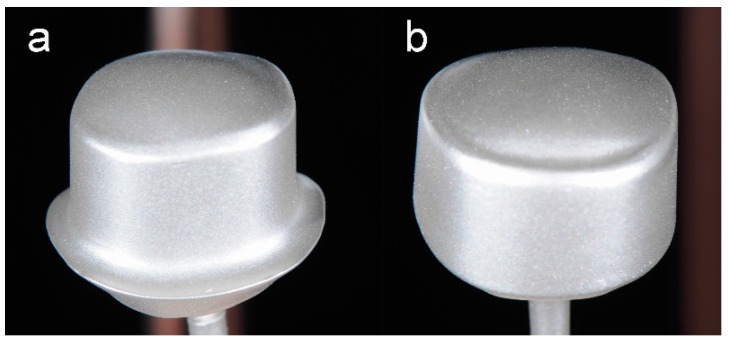
Primary crowns of (**a**) 0°; and (**b**) 2° on copper sticks, air brushed with silver conductive lacquer.

The electroforming process lasted 14 h, applying 17 mA voltage per crown. For this process, the inner surface of the finished detached primary crowns (30 × Y-TZP + 30 × CoCr) was air-abraded and cleaned. Then, the two components of polyurethane resin (PU; Helioform Polyurethane material compount A & B; C. Hafner GmbH & Co. KG, LOT: 512) were mixed and agitated in a 1:1 ratio for 30 s, filled into the primary crowns and hardened for 30 min. These PU-auxiliary parts were combined with copper stickers resulting in the anode. For accumulation of gold ions the silver conductive lacquer presented the guide rail. Using the air-brush gun allowed an even, thin coating of silver conductive lacquer (Helioform silver conductive spacer for airbrush; C. Hafner GmbH & Co. KG, LOT: 02/13) on the surface area of the primary crowns (Figure 3). A wider track of silver conductive lacquer (Helioform silver conductive spacer; C. Hafner GmbH & Co. KG, LOT: 02/13) was necessary to connect the surface area with the copper anode. The bottom and the fringe area were covered with a light-curing cover lacquer (Helioform cover varnish LC; C. Hafner GmbH & Co. KG, LOT: 122574) to prevent electroforming at these areas.

**Figure 4:**
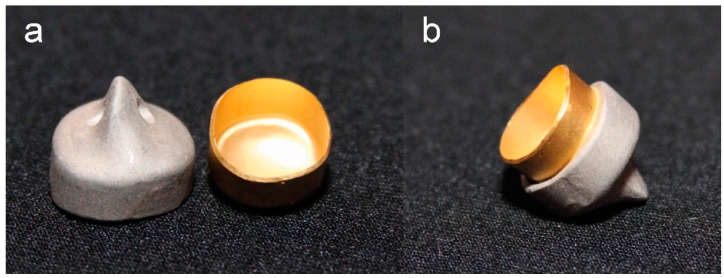
Electroformed gold coping and CoCr tertiary structure, separate (**a**); and assembled (**b**).

To prevent a plastic deformation, the delicate gold copings were pasted into a CoCr tertiary structure (AGC Cem Automix system, Wieland Dental GmbH & Co. KG, LOT: 697720) (Figure 4).

### 2.4. Statistical Analyses

The maximum retention load values of each assembly were used for descriptive statistics, including mean, standard deviation (SD), 95% confidence interval (CI), minimum, median and maximum values. Furthermore, verification of data normality distribution was executed by Kolmogorov-Smirnov and Shapiro-Wilk tests. Significant differences in maximum retention load between the groups were detected by one-way and two-way ANOVA, ensured by the *post-hoc* Scheffé test. IBM SPSS (Version 22; IBM Corporation) was basic for the statistical tests with *p* < 0.05 as the significant level.

## 3. Results

With regard to the taper, Y-TZP secondary crowns with 1° on CoCr primary crowns showed significantly higher (*p* = 0.009) retention load values compared to those of 0° and 2°. Electroformed copings on CoCr with 1° also showed significantly higher (*p* = 0.001) retention load values than with 0° and 2°. In addition, secondary crowns on Y-TZP primary crowns showed no significant differences in retention load regarding the taper (Z/Z: *p* = 0.167; Z/G: *p* = 0.069) (Table 1).

Concerning the fabrication method and disregarding the primary crowns, electroformed secondary crowns with 0° showed significantly lower retention load values than secondary crowns made of Y-TZP (*p* < 0.001). Other than that, the primary crowns have no significant impact on the retention load within the 0° taper group. Z/G showed significantly lower retention load values compared to the C/Z within the 1° taper group (*p* = 0.007) and Z/Z in the 2° taper group (*p* = 0.006).

## 4. Discussion

This study examined the retention loads of different telescopic crown systems of three different tapers, *i.e.*, 0° with a chamfer and 1° and 2° with a tangential ending, respectively. Two different materials were used as primary crowns: Y-TZP and CoCr. Both were CAD/CAM-milled and later on sintered and they displayed comparable elastic moduli. Each primary crown was coupled with a Y-TZP secondary crown and an electroformed coping.

The first null hypothesis regarding the taper angle was rejected since the two telescopic crown systems C/Z and C/G with 1° showed significantly higher retention load values as compared to those with 0° and 2°. Basically, the design of the primary crown could have an influence on the retention load values as shown in an earlier study [19]. According to Beuer and co-workers, 0° telescopic crowns need a chamfer design to create adhesion, but this has been shown to negatively influence the retention forces [19].

Our result seems to be in contrast to the matter of common knowledge based on Ohkawa *et al.* and other studies [9,20,21], who have shown that an increasing retention load occurs with decreasing taper angles. This statement is based on investigating taper angles from 0° to 6°. Turp *et al.* [9] emphasized that a statistically significant difference occurred only with a difference of more than 2° in taper angle. This result was corroborated by our findings, namely by the groups Z/Z and Z/G, which showed no significant differences in retention load within the three taper angle groups of 0°, 1° and 2°. However, Güngör recommended that the taper angles should not exceed more than 2° in case of long-term use [20]. Higher retention load values for 1° have been confirmed in the literature recently [22]. The authors examined the range from 0° to 2° and also observed higher retention load values for 1° telescopic crowns.

In addition, the second null hypothesis was accepted because no significant differences could be found between groups with different primary crowns and the same secondary crown types (C/Z and Z/Z; C/G and Z/G). In the present study the two primary crown materials had similar elastic moduli (204 GPa for Y-TZP and 200 GPa or greater for CoCr). Already in 1975, Garvie characterized a non-precious metal alloy and ceramic with his description of ZrO_2_ as “ceramic steel” [23]. Appertaining to that result, Besimo and co-workers stated in 1996 that the retention force of telescopic crowns is not significantly affected by the primary crown material [18].

In contrast to Besimo, Beuer *et al.* observed in 2010 that the surface roughness of the primary crowns affects the retention force of electroformed assemblies [19]. In their study, the Y-TZP primary crowns yielded higher retention load values with smoother surfaces. This interrelationship was explained by a smaller gap between the primary crown and coping [19], which can be achieved by a smoother surface after grinding and polishing [16]. In a recent study it was stated that friction generally depends on the specific surface roughness (R_a_) of the materials (ZrO_2_: R_a_ = 0.02 µm, CoCr: R_a_ = 0.44 µm) [24].

In the present study, Y-TZP and electroformed gold copings were used for secondary crowns. In this context, we found that groups with electroformed copings resulted in significantly lower retention load values as compared to Y-TZP groups, especially in the 0° taper configuration. This result is in accordance with recent studies, in which the combination of Y-TZP primary crown with an electroformed gold coping showed lower, more predictable and less excursive retention loads than conventionally cast telescopic crowns [9,14]. In another study, the galvanic copings yielded a better fit in comparison to casted ones [16]. The reason for this can be the small gap between the functional surfaces of the telescopic crown [16]. The automatic electroplating process achieves a smooth internal coping surface [16] and does not require any manually performed retention load adjustment [15].

Unfortunately, there exists no universal guideline for investigating telescopic crowns yet. Even the presence of saliva influences the results and increases retention load values [24]. Therefore, in the experimental setup, artificial saliva was used in all groups and each telescopic crown assembly was preloaded with 50 N as presented in literature [9,19,21]. Nevertheless, in this study, initial values were investigated. Advanced research about thermo-mechanical aging is necessary. Further limitations of the study are the lack of fatigue and clinical testing.

## 5. Conclusions

Considering the different taper angles, significant differences in two of four groups (C/Z, C/G) can only be noticed in the 1° group, evoked by the interaction of different materials and the design parameters of 1°. With regard to the two primary crown materials, Y-TZP and CoCr, no significant differences of retention loads can be observed. If significant statistical differences occurred, electroformed copings showed lower retention load values compared to Y-TZP secondary crowns in each taper group.

## Figures and Tables

**Table 1:** Descriptive statistics such as mean with standard deviation (SD), 95% confidence intervals (95% CI) and the non-parametric statistics (minimum/median/maximum). All values are presented in Newton (N).

Taper Angle	Assemblies	Mean ± SD	95% CI	Min/Median/Max
0°	C/Z	17.38 ± 6.98	(12.2; 22.4)	7.6/14.9/29.6
	Z/Z	17.63 ± 5.16	(13.8; 21.4)	6.4/18.0/24.0
	C/G	10.38 ± 2.85	(8.2; 12.5)	4.8/10.1/14.0
	Z/G	7.73 ± 5.37	(3.7; 11.6)	1.1/7.7/15.0
1°	C/Z	26.44 ± 5.48	(22.4; 30,4)	15.9/27.4/34.1
	Z/Z	17.92 ± 6.92	(12.8; 22.9)	8.4/16.5/30.0
	C/G	22.40 ± 8.73	(16.0; 28.7)	12.5/20.3/37.0
	Z/G	14.63 ± 8.26	(8.6; 20.6)	3.1/12.1/29.0
2°	C/Z	16.86 ± 8.59	(10.6; 23.1)	5.1/18.3/31.4
	Z/Z	22.71 ± 7.31	(17.3; 28.0)	16.4/18.4/35.4
	C/G	14.74 ± 6.05	(10.3; 19.1)	4.2/14.3/25.9
	Z/G	11.35 ± 4.87	(7.7; 14.9)	5.3/10.2/20.5

C/Z: CoCr primary crown, Y-TZP secondary crown; Z/Z: Y-TZP primary crown, Y-TZP secondary crown; C/G: CoCr primary crown, electroformed gold coping; Z/G: Y-TZP primary crown, electroformed gold coping.

## Abbreviations

The following abbreviations are used in this manuscript:

ZrO_2_	Zirconia
Y-TZP	Yttrium-stabilized tetragonal zirconium oxide
CoCr	Cobalt-chromium alloy
RL	Retention load
CAD/CAM	Computer-aided design/computer-aided manufacturing
FDPs	Fixed dental prostheses
RDPs	Removable partial dentures

## References

[1] Manicone P.F., Rossi Iommetti P., Raffaelli L. (2007). An overview of zirconia ceramics: Basic properties and clinical applications. J. Dent..

[2] Möller B., Terheyden H., Açil Y., Purcz N.M., Hertrampf K., Tabakov A., Behrens E., Wiltfang J. (2012). A comparison of biocompatibility and osseointegration of ceramic and titanium implants: An *in vivo* and *in vitro* study. Int. J. Oral Maxillofac. Surg..

[3] Piconi C., Maccauro G. (1999). Zirconia as a ceramic biomaterial. Biomaterials.

[4] Ferrari M., Sorrentino R., Cagidiaco C., Goracci C., Vichi A., Gherlone E., Zarone F. (2015). Short-term clinical performance of zirconia single crowns with different framework designs: 3-year clinical trial. Am. J. Dent..

[5] Chaar M.S., Passia N., Kern M. (2015). Ten-year clinical outcome of three-unit posterior FDPs made from a glass-infiltrated zirconia reinforced alumina ceramic (In-Ceram Zirconia). J. Dent..

[6] Thoma D.S., Brandenberg F., Fehmer V., Knechtle N., Hämmerle C.H., Sailer I. (2015). The Esthetic Effect of Veneered Zirconia Abutments for Single-Tooth Implant Reconstructions: A Randomized Controlled Clinical Trial. Clin. Implant. Dent. Relat. Res..

[7] Groesser J., Sachs C., Heiß P., Stadelmann M., Erdelt K., Beuer F. (2014). Retention forces of 14-unit zirconia telescopic prostheses with six double crowns made from zirconia—An *in vitro* study. Clin. Oral Investig..

[8] Näpänkangas R., Pihlaja J., Raustia A. (2015). Outcome of zirconia single crowns made by predoctoral dental students: A clinical retrospective study after 2 to 6 years of clinical service. J. Prosthet. Dent..

[9] Turp I., Bozdağ E., Sünbüloğlu E., Kahruman C., Yusufoğlu I., Bayraktar G. (2014). Retention and surface changes of zirconia primary crowns with secondary crowns of different materials. Clin. Oral Investig..

[10] Guess P.C., Bonfante E.A., Silva N.R., Coelho P.G., Thompson V.P. (2013). Effect of core design and veneering technique on damage and reliability of Y-TZP-supported crowns. Dent. Mater..

[11] Schmitter M., Lotze G., Bömicke W., Rues S. (2015). Influence of surface treatment on the *in-vitro* fracture resistance of zirconia-based all-ceramic anterior crowns. Dent. Mater..

[12] Kolgeci L., Mericske E., Worni A., Walker P., Katsoulis J., Mericske-Stern R. (2014). Technical complications and failures of zirconia-based prostheses supported by implants followed up to 7 years: A case series. Int. J. Prosthodont..

[13] Larsson C., Vult von Steyern P. (2016). Ten-Year Follow-Up of Implant-Supported All-Ceramic Fixed Dental Prostheses: A Randomized, Prospective Clinical Trial. Int. J. Prosthodont..

[14] Engels J., Schubert O., Güth J.F., Hoffmann M., Jauernig C., Erdelt K., Stimmelmayr M., Beuer F. (2013). Wear behavior of different double-crown systems. Clin. Oral Investig..

[15] Bayer S., Kraus D., Keilig L., Gölz L., Stark H., Enkling N. (2012). Wear of double crown systems: Electroplated *vs.* casted female part. J. Appl. Oral Sci..

[16] Weigl P., Hahn L., Lauer H.C. (2000). Advanced biomaterials used for a new telescopic retainer for removable dentures. J. Biomed. Mater. Res..

[17] Goodfellow. http://www.goodfellow.com/G/Gold.html.

[18] Besimo C.H., Graber G., Flühler M. (1996). Retention force changes in implant-supported titanium telescope crowns over long-term use *in vitro*. J. Oral Rehabil..

[19] Beuer F., Edelhoff D., Gernet W., Naumann M. (2010). Parameters affecting retentive force of electroformed double-crown systems. Clin. Oral Investig..

[20] Güngör M.A., Artunç C., Sonugelen M. (2004). Parameters affecting retentive force of conus crowns. J. Oral Rehabil..

[21] Ohkawa S., Okane H., Nagasawa T., Tsuru H. (1990). Changes in retention of various telescope crown assemblies over long-term use. J. Prosthet. Dent..

[22] Wagner C., Stock V., Merk S., Schmidlin P.R., Roos M., Eichberger M., Stawarczyk B. (2015). Comparison of retention forces of different fabrication methods of Co-Cr crowns: Presintered and milled, cast and electroforming secondary crowns with different taper angles. Int. J. Dentistry Oral Sci..

[23] Garvie R.C., Hannink R.H., Pascoe R.T. (1975). Ceramic steel?. Nature.

[24] Dąbrowa T., Dobrowolska A., Wieleba W. (2013). The role of friction in the mechanism of retaining the partial removable dentures with double crown system. Acta Bioeng. Biomech..

